# Global and Regional Burden of Type 2 Diabetes Mellitus Attributable to Low Physical Activity From 1990 to 2021

**DOI:** 10.1111/1753-0407.70043

**Published:** 2025-01-06

**Authors:** Lihang Yang, Diya Xie, Fengmin Liu, Jiaying Lin, Xin Lin, Yuquan Chen, Kun Zhang

**Affiliations:** ^1^ Department of Endocrinology Fuzhou First General Hospital Affiliated With Fujian Medical University Fuzhou Fujian China; ^2^ Department of General Surgery Fuzhou First General Hospital Affiliated With Fujian Medical University Fuzhou Fujian China; ^3^ Department of Endocrinology Fujian Maternity and Child Health Hospital Affiliated Hospital of Fujian Medical University Fuzhou Fujian China; ^4^ Department of Endocrine and Metabolic Diseases, Shanghai Institute of Endocrine and Metabolic Diseases, Ruijin Hospital Shanghai Jiao Tong University School of Medicine Shanghai China; ^5^ Department of Endocrinology Mengchao Hepatobiliary Hospital of Fujian Medical University Fuzhou Fujian China

**Keywords:** disability‐adjusted life years, global burden, low physical activity, mortality, sociodemographic index, type 2 diabetes mellitus

## Abstract

**Aim:**

Type 2 diabetes mellitus (T2DM) contributes to the heavy burden, but there lacks latest and comprehensive global research on the burden of T2DM attributable to low physical activity (LPA). This study aimed to quantify the global and regional burden of T2DM attributable to LPA from 1990 to 2021.

**Methods:**

We utilized data including disability‐adjusted life years (DALYs), mortality, age‐standardized disability‐adjusted life years (ASDR), and age‐standardized mortality rates (ASMR) from the Global Burden of Disease (GBD) 2021. We assessed the burden across different ages, genders, and sociodemographic index (SDI). Joinpoint regression analysis was applied to estimated average annual percent change (AAPC).

**Results:**

Between 1990 and 2021, DALYs and mortality of T2DM attributable to LPA increased rapidly. There was an increase in the ASDR and ASMR, with AAPC of 1.09 (95% CI: 1.03–1.16) and 0.32 (95% CI: 0.2–0.43), which was increased faster in males. Low‐middle SDI countries have the highest ASDR and highest ASMR. The global PAF for ASDR and ASMR in 2021 is 7.38% and 9.45%. A U‐shaped drift pattern was observed in most SDI quintiles in APC model. Population growth is a major contributor to the burden of T2DM, especially in countries with low SDI. Epidemiological changes also play an important role in DALYs and mortality. A negative correlation existed between SDI and both ASMR and ASDR.

**Conclusion:**

Between 1990 and 2021, there was a marked rise in the global burden of T2DM associated with LPA. The findings lay the groundwork for informed decision‐making a public health and healthcare delivery.

**Summary**

- Based on the Global Burden of Disease Study (GBD), we found that there was a marked rise in the global burden of type 2 diabetes mellitus (T2DM) attributable to low physical activity (LPA).
- LPA is a significant risk factor for T2DM and varied in different genders and different regions.
- Drafting impactful public health strategies which increase the level of physical activity (PA) can decrease the burden of T2DM globally.

## 1 Introduction

Type 2 diabetes mellitus (T2DM) is a chronic metabolic disorder characterized by insulin resistance, relative insulin deficiency, and hyperglycemia [1]. It has become a pandemic health issue, with its burden increasing in both developed and developing countries, affecting millions of individuals worldwide and imposing a substantial burden on healthcare systems. The disease not only impairs the quality of life for those affected, but also significantly contributes to the global burden of noncommunicable disease [2]. T2DM may causes various complications, including cardiovascular diseases (CVD), kidney failure, blindness, and lower limb amputations [3]. The economic impact of T2DM is substantial, with direct medical costs and indirect costs due to disability and premature mortality. The management of T2DM and its complications places a heavy burden on individuals, families, and societies.

Understanding the epidemiology of T2DM and its risk factors is crucial for developing effective prevention and management strategies. While genetic predisposition plays a role in the development of T2DM, lifestyle factors, such as poor diet and sedentary behavior, have been identified as significant modifiable risk factors [4, 5]. Among these, low physical activity (LPA) has emerged as a key contributor to the pathogenesis of T2DM. Previous study found that there was a marked rise in the worldwide burden of T2DM associated with physical inactivity between 1990 and 2019 from the Global Burden of Disease Study (GBD) database [6, 7]. LPA influences the pathogenesis of T2DM through multiple interconnected pathways that promote inflammation, dyslipidemia, and endothelial dysfunction. These mechanisms collectively contribute to insulin resistance and β‐cell dysfunction [8, 9]. Therefore, enhancing physical activity (PA) is critical in the prevention and management of T2DM and its associated complications [10]. With the open of the latest 2021 version of GBD database [11], this study aims to provide an updated overview of the global and regional epidemiology of T2DM, with a particular focus on the role of LPA as a risk factor and find its trend.

## 2 Methods

### 2.1 Data Source and Definitions

This study utilized data from GBD 2021, which provides comprehensive estimates for 371 diseases and injuries across 204 countries and territories from 1990 to 2021 [12]. T2DM in the GBD study is defined based on the International Classification of Diseases, Tenth Edition (ICD‐10), including cases with fasting plasma glucose levels above 126 mg/dL or those receiving treatment for T2DM [13]. To accurately attribute mortality to T2DM due to LPA, we employed the ICD‐10 codes to distinguish deaths directly attributable to T2DM from those attributable to CVD. We utilized E11–E14 codes for T2DM and excluded codes related to CVD including I20–I25, I60–I69, and I70–I79 to ensure that the reported mortality were specifically due to T2DM and not confounded by CVD. LPA is defined as engaging in less than 3000–4500 metabolic equivalent (MET) minutes per week across all domains of activity, such as occupational, household, transport, and leisure activities [14]. The study focused on key metrics including disability‐adjusted life years (DALYs) and mortality (deaths), age‐standardized disability‐adjusted life year rates (ASDR), and age‐standardized mortality rates (ASMR) associated with T2DM attributable to LPA [15]. Rates and ASRs were computed for every 100 000 individuals, accompanied by 95% uncertainty intervals (UIs). The study also employed the sociodemographic index (SDI), a composite measure of socioeconomic development, to examine T2DM burden across different levels of socioeconomic status, categorizing countries into five SDI quintiles. The study was conducted in accordance with the Accurate and Transparent Health Estimates Reporting (GATHER) statement [16]. Details for ASR and SDI were provided in Supporting Information.

### 2.2 Joinpoint Regression Analysis

In this study, Joinpoint regression analysis was applied to evaluate temporal trends in ASDR and ASMR associated with T2DM attributable to LPA from 1990 to 2021. This method is particularly advantageous because it allows for the identification of multiple trend changes (joinpoints) over the study period, offering a more nuanced understanding of how rates have evolved over time. By fitting a series of joined straight lines to the logarithmic transformation of the rates, the model captures both global trends and local variations. The grid search method (GSM) [17] was used to determine the optimal number and location of joinpoints, while the Monte Carlo permutation test was employed for model optimization, ensuring that the identified trends are statistically robust. The annual percent change (APC) for each segment was determined to ascertain the directionality and velocity of the trends observed. Furthermore, the average annual percent change (AAPC) was employed to distill the general trend across all segments, representing a weighted mean of the APCs. The APC and AAPC are articulated as percentages (%), each accompanied by a 95% confidence interval (CI) to reflect the statistical robustness of the trend estimates. A positive APC and AAPC (> 0%) are indicative of an upward trend, whereas a negative value (< 0%) denotes a downward trend. A 95% CI that spans zero implies a stable trend over the period in question. Details were provided in Supporting Information.

### 2.3 Population Attributable Fractions (PAFs)

PAFs for T2DM attributable to LPA were estimated to quantify the proportion of the disease burden that could be avoided if LPA were eliminated. PAFs were calculated by comparing the current exposure levels to a theoretical minimum risk exposure level, assuming all other factors remained constant. The PAFs were then multiplied by the total burden of T2DM (in terms of ASDR and ASMR) to estimate the burden for different SDI quintiles, regions, and genders in 2021.

### 2.4 Age‐Period‐Cohort (APC) Model

The APC model is a sophisticated epidemiological tool renowned for its ability to address the issue of collinearity among age, period, and cohort effects, which are often interdependent [18]. This model stands out for its capacity to independently assess the effects of age, period, and cohort, a feature that is particularly advantageous in resolving the statistical challenges posed by their inherent correlation. Our analysis spanned ages from 25 to 95+ and years from 1992 to 2021, with 5‐year intervals. Utilizing the APC web tool from the National Cancer Institute [19], with the reference age set at 40 and the reference year at 2002, we estimated the five key parameters including (a) net drift, which represents the overall annual percentage change in age‐standardized rates, reflecting the general temporal trend in disease incidence or mortality; (b) local drifts, quantifying the model‐based estimated annual percentage change specific to each age group, highlighting variations in temporal trends across different ages; (c) longitudinal age curve (age effect), describing the natural history of disease associated with aging, adjusted for period and cohort effects, providing insights into age‐specific risks; (d) period rate ratios (RRs), comparing disease rates across calendar periods, adjusted for age and cohort effects, to identify time‐specific changes in disease occurrence; and (e) cohort rate ratios, assessing the relative disease rates across different birth cohorts, adjusted for age and period effects, revealing generational differences in disease risk. Details and the interpretation of indicators were provided in Supporting Information.

### 2.5 Decomposition Analysis

We employed a decomposition analysis of Das Gupta [20] to gain an in‐depth understanding of the factors driving the changes in the global burden of T2DM attributable to LPA from 1990 to 2021. By decomposing the DALYs and mortality into disease subgroups, sexes, population size, age structure, and epidemiological changes, we were able to quantify the contribution of each factor to the changes. Specifically, we utilized a formula that considers the proportion of the population in different age categories, the total population, and the DALYs rate for specific age groups to calculate the DALYs for each year. This approach allowed us to assess the impact of population growth, aging, and epidemiological changes on the epidemiology of T2DM while holding other factors constant. The utility of this analysis lies in its ability to not only reveal the underlying causes of changes in the burden of T2DM, but also to inform the development of targeted public health interventions. Formula was provided in Supporting Information.

### 2.6 Correlation Analysis

Correlation analysis [21] were conducted to explore the relationship between SDI values and ASDR, ASMR, and PAFs for T2DM attributable to LPA. Spearman correlation coefficients (*ρ*) were calculated to assess the strength and direction of these associations, providing insights into the influence of socioeconomic development on the burden of T2DM linked to LPA.

Data for the study were sourced from the Global Health Data Exchange (GHDx) query tool (http://ghdx.healthdata.org/gbd‐results‐tool). All statistical analyses were conducted using R software (v4.4.1), with visualizations created using the ggplot2 package. Statistical significance was determined at a *p* value threshold of less than 0.05.

## 3 Results

### 3.1 Main Findings: Global Burden of LPA and T2DM

#### 3.1.1 Global Burden of T2DM Attributable to LPA and Trends in ASDR and ASMR

The global DALY count for T2DM attributable to LPA has seen a substantial increase from 1 755 081.59 (95% UI: 756 858.62–2 694 173.44) in 1990 to 5 523 050.32 (95% UI: 2 407 128.15–8 638 534.85) in 2021, reflecting an approximate 3.13‐fold increase (Table 1). The mortality cases due to T2DM attributable to LPA also surged, increasing from 55 801 (95% UI: 24 048.92–85 577.17) in 1990 to 149 213.77 (95% UI: 65 193.59–228 317.89) in 2021, an approximate 2.68‐fold increase (Table 2).

**TABLE 1:** DALYs for T2DM attributed to LPA: age‐standardized rates with 95% uncertainty intervals and annual percentage change (AAPC) with 95% confidence Intervals, 1990–2021.

	1990		2021		1990–2021
	DALYs (disability‐adjusted life years)	ASDR per 100 000	DALYs (disability‐adjusted life years)	ASDR per 100 000	AAPC
	No. ×10^5^ [95% UI]	No. [95% UI]	No. ×10^5^ [95% UI]	No. [95% UI]	% [95% CI]
Global	17.55[7.57–26.94]	46.06 [19.90–70.69]	55.23 [24.07–86.39]	64.27 [28.01–100.49]	1.09 [1.03–1.16]
Female	11.33[4.96–17.51]	53.94 [23.65–83.42]	34.28 [14.87–53.31]	74.38 [32.27–115.78]	1.04 [0.99–1.09]
Male	6.22[2.65–9.74]	36.48 [15.55–56.64]	20.95 [9.07–32.91]	52.92 [22.89–83.10]	1.21 [1.14–1.29]
High SDI	4.32[1.87–6.70]	38.79 [16.84–60.23]	10.58 [4.70–16.97]	52.10 [23.03–84.44]	0.95 [0.78–1.13]
High‐middle SDI	3.49[1.51–5.34]	36.66 [15.98–56.34]	9.51 [4.16–14.92]	48.14 [21.04–75.47]	0.89 [0.75–1.02]
Middle SDI	5.49[2.38–8.47]	56.85 [24.60–88.05]	20.07 [8.77–31.17]	75.81 [33.18–117.39]	0.94 [0.84–1.04]
Low‐middle SDI	3.19[1.37–4.91]	56.90 [24.41–87.64]	12.04 [5.20–18.71]	88.59 [38.29–137.73]	1.44 [1.26–1.63]
Low SDI	1.04[0.43–1.62]	50.22 [20.71–78.34]	2.96 [1.25–4.70]	62.74 [26.40–99.23]	0.72 [0.62–0.83]

Abbreviations: 95% CI, 95% confidential intervals; AAPC, average annual percent change; ASDR, age‐standardized disability‐adjusted life years rate; DALYs, disability‐adjusted life years; LPA, low physical activity; SDI, sociodemographic index; T2DM, type 2 diabetes mellitus; UI, uncertainty interval.

**TABLE 2:** Mortality for T2DM attributed to LPA: age‐standardized rates with 95% uncertainty intervals and annual percentage change (AAPC) with 95% confidence Intervals, 1990–2021.

	1990		2021		1990–2021
	Mortality	ASMR per 100 000	Mortality	ASMR per 100 000	AAPC
	No. ×10^5^ [95% UI]	No. [95% UI]	No. ×10^5^ [95% UI]	No. [95% UI]	% [95% CI]
Global	0.56 [0.24–0.86]	1.64 [0.71–2.51]	1.49 [0.65–2.28]	1.80 [0.79–2.75]	0.32 [0.20–0.43]
Female	0.38 [0.17–0.58]	1.89 [0.85–2.91]	0.95 [0.42–1.45]	2.03 [0.90–3.11]	0.25 [0.11–0.39]
Male	0.18 [0.07–0.28]	1.28 [0.53–1.95]	0.54 [0.23–0.84]	1.50 [0.65–2.34]	0.53 [0.35–0.72]
High SDI	0.15 [0.06–0.23]	1.31 [0.56–2.02]	0.21 [0.09–0.33]	0.88 [0.37–1.37]	−1.28 [−1.52 to −1.04]
High‐middle SDI	0.11 [0.05–0.17]	1.28 [0.56–1.95]	0.25 [0.11–0.39]	1.30 [0.58–1.98]	0.09 [−0.18 to 0.36]
Middle SDI	0.16 [0.07–0.25]	2.03 [0.87–3.13]	0.56 [0.25–0.84]	2.32 [1.03–3.53]	0.46 [0.28–0.65]
Low‐middle SDI	0.10 [0.04–0.16]	2.19 [0.93–3.34]	0.38 [0.16–0.58]	3.22 [1.39–4.96]	1.30 [0.97–1.62]
Low SDI	0.04 [0.01–0.05]	2.04 [0.83–3.19]	0.09 [0.04–0.14]	2.33 [0.96–3.70]	0.44 [0.23–0.66]

Abbreviations: 95% CI, 95% confidential intervals; AAPC, average annual percent change; ASMR, age‐standardized mortality rate; LPA, low physical activity; SDI, sociodemographic index; T2DM, type 2 diabetes mellitus; UI, uncertainty interval.

**FIGURE 1:**
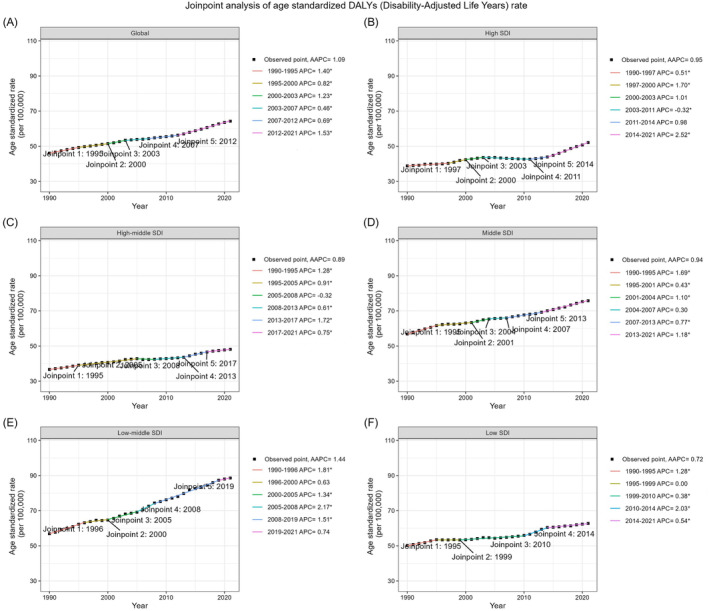
Global trends in ASDR for T2DM attributable to LPA and across five SDI quintiles from 1990 to 2021. AAPC, average annual percent change; APC, annual percent change; ASDR, age‐standardized disability‐adjusted life years rate; LPA, low physical activity; SDI, sociodemographic index; T2DM, type 2 diabetes mellitus.

**FIGURE 2:**
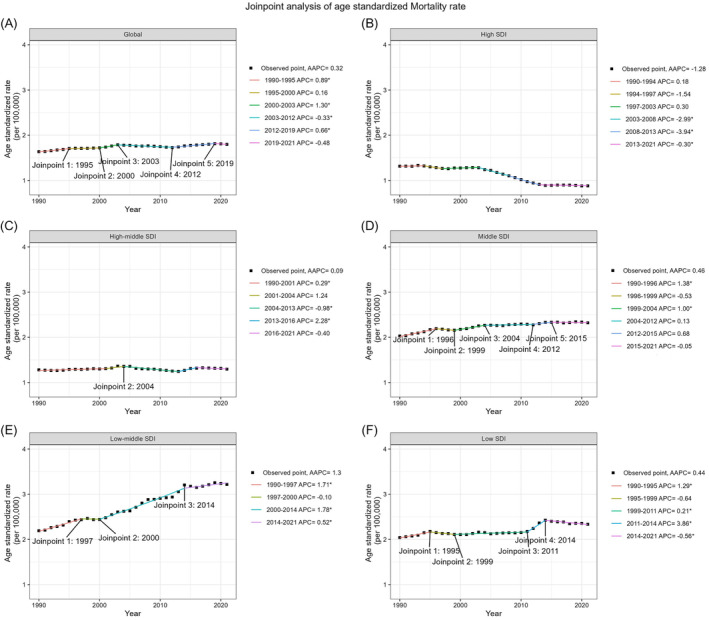
Global trends in ASMR for T2DM attributable to LPA and across five SDI quintiles from 1990 to 2021. AAPC, average annual percent change; APC, annual percent change; ASMR, age‐standardized mortality rate; LPA, low physical activity; SDI, sociodemographic index; T2DM, type 2 diabetes mellitus.

The ASDR exhibited an upward trend with an AAPC of 1.09 (95% CI: 1.03–1.16) (Figure 1). The ASMR per 100 000 people saw a modest rise, with an AAPC of 0.32% (95% CI: 0.20–0.43) (Figure 2).

#### 3.1.2 Sex and SDI Disease Burden

As shown in Table 1, stratified by gender, females showed an increase in ASDR from 53.94 (95% UI: 23.65–83.42) in 1990 to 74.38 (95% UI: 32.27–115.78) in 2021, with an AAPC of 1.04 (95% CI: 0.99–1.09). Males experienced a higher rate of increase, with ASDR rising from 36.48 (95% UI: 15.55–56.64) in 1990 to 52.92 (95% UI: 22.89–83.10) in 2021, and an AAPC of 1.21 (95% CI: 1.14–1.29). As shown in Table 2, for mortality, the ASMR for females increased from 1.89 (95% UI: 0.85–2.91) in 1990 to 2.03 (95% UI: 0.90–3.11) in 2021, with an AAPC of 0.25% (95% CI: 0.11–0.39). Males' ASMR rose from 1.28 (95% UI: 0.53–1.95) to 1.50 (95% UI: 0.65–2.34), with an AAPC of 0.53% (95% CI: 0.35–0.72).

The low‐middle SDI quintile reported the highest ASDR in 2021 at 88.59 (95% UI: 38.29–137.73), with the steepest rising, an AAPC of 1.44 (95% CI: 1.26–1.63). The high SDI quintile showed the fastest rise in recent years with an annual percentage change (APC) from 2014 to 2021 at 2.52 (95% CI: 2.38–2.67) (Table 1). For mortality, the high SDI quintile was the only one to note a decrease in ASMR, with a significant AAPC of −1.28% (95% CI: −1.52 to −1.04), while the low‐middle SDI quintile had the highest ASMR in 2021, with an AAPC of 1.30% (95% CI: 0.97–1.62) (Table 2).

### 3.2 PAFs of T2DM Attributable to LPA

**FIGURE 3:**
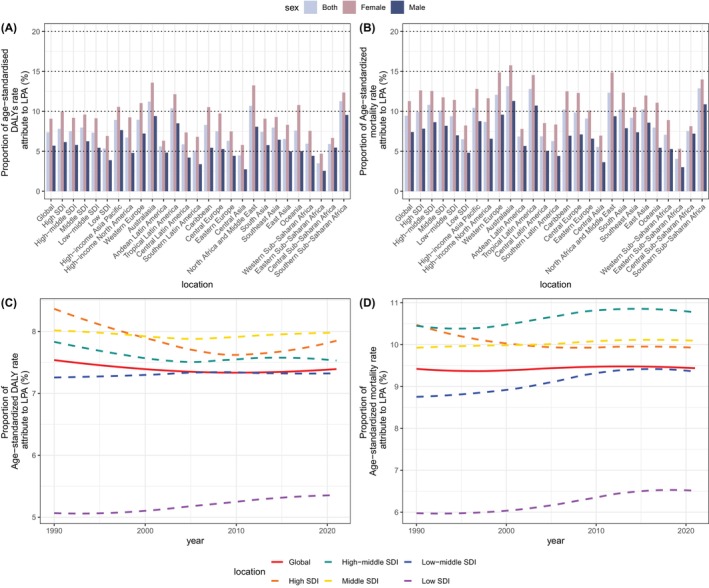
Global and five SDI quintiles trends in T2DM burden attributable to LPA from 1990 to 2021. (A) PAFs for ASDR and (B) ASMR of T2DM associated with LPA in 2021. (C) Temporal trends in PAFs for ASDR and (D) ASMR of T2DM globally and by SDI quintiles due to LPA, 1990–2021. ASDR, age‐standardized disability‐adjusted life years rate; ASMR, age‐standardized mortality rate; DALYs, disability‐adjusted life years; LPA, low physical activity; PAF, population attributable fractions; SDI, sociodemographic index; T2DM, type 2 diabetes mellitus.

In 2021, the global PAFs for ASDR and ASMR were 7.38% (95% CI: 3.16%–11.35%) and 9.45% (95% CI: 4.05%–14.29%), respectively. Notably, the PAFs for ASDR reached their peak in the middle SDI quintile at 7.97% (95% CI: 3.43%–12.01%), whereas for ASMR, the high‐middle SDI quintile reported the highest PAF at 10.81% (95% CI: 4.83%–16.39%) (Figure 3, Table S1). In contrast, the lowest PAFs for both ASDR and ASMR were observed in the low SDI quintile, with 5.35% (95% CI: 2.22%–8.19%) and 6.51% (95% CI: 2.67%–9.92%), respectively, highlighting the significant impact of socioeconomic status (SES) on the health effects of LPA.

Over the 31‐year period from 1990 to 2021, there has been a shift of PAFs for ASDR in the high SDI quintile, which moved from the leading position to the second rank, while the low SDI quintile has shown a continuous rise. For ASMR, high‐middle SDI kept the highest PAF from 1990 to 2021 and high SDI presented a slightly decreasing trend. Gender disparities are consistent, with female PAFs generally exceeding those of males for both ASDR and ASMR. This pattern suggests a marginally greater influence of LPA on the T2DM burden among females across different regions.

### 3.3 APC Model

**FIGURE 4:**
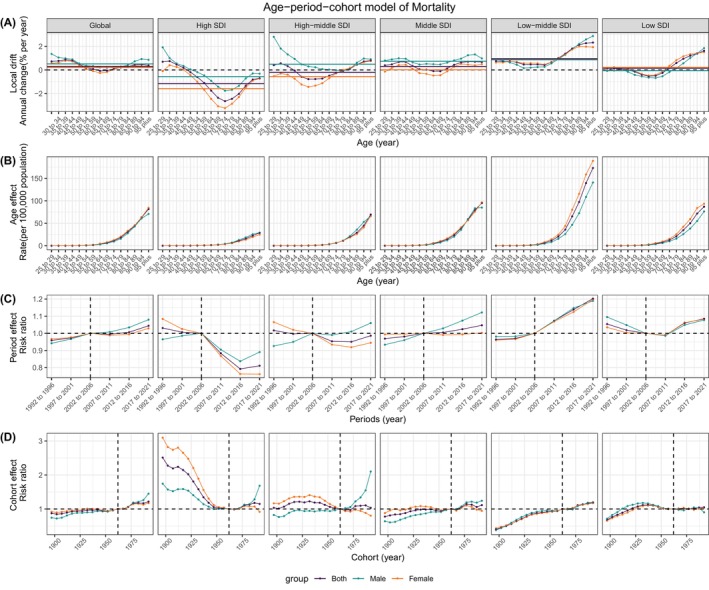
Estimates of age, period, and cohort effects on DALYs due to T2DM attributed to LPA globally and in different SDI regions. (A) Local drift, the horizontal‐colored lines represent the net drift of different groups; (B) age effect; (C) period effect; (D) cohort effect. DALYs, disability‐adjusted life years; LPA, low physical activity; SDI, sociodemographic index; T2DM, type 2 diabetes mellitus.

**FIGURE 5:**
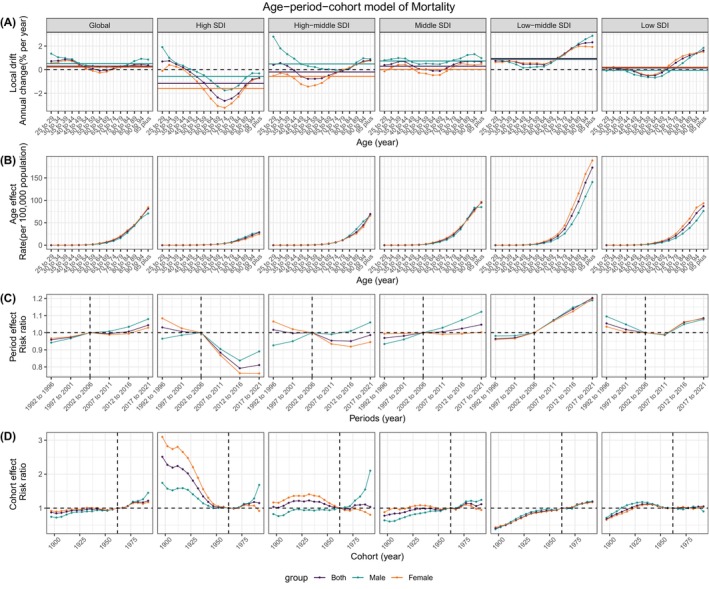
Estimates of age, period, and cohort effects on mortality due to T2DM attributed to LPA globally and in different SDI regions. (A) Local drift, the horizontal‐colored lines represent the net drift of different groups; (B) age effect; (C) period effect; (D) cohort effect. LPA, low physical activity; SDI, sociodemographic index; T2DM, type 2 diabetes mellitus.

APC model was applied to dissect the impact of age, period, and cohort on the DALYs (Figure 4) and mortality (Figure 5) of T2DM attributable to LPA. The net drift in DALYs was positive across all SDI quintiles, corroborating our previous findings of a rise in DALYs over the 31‐year period. A U‐shaped local drift pattern was observed in most SDI quintiles, with the nadir shifting to older ages as SDI increased.

This pattern indicated a general increase in DALYs, with a more marked rise in younger age groups. Notably, in the high, high‐middle, and middle SDI quintiles, males exhibited a higher local drift in DALYs compared to females, an inverse trend was observed in the low SDI quintiles. Mortality trends revealed a negative net drift in the high and high‐middle SDI quintiles, contrasting with a positive net drift in the remaining quintiles. Particularly in the high SDI quintile, the 70–74 years age group demonstrated the lowest local drift, signaling a decline in mortality for this demographic. After the age of 70–74 years, mortality rates continued to escalate with advancing age, with the most notable increase observed in the low‐middle SDI and low SDI quintiles.

### 3.4 Decomposition Analysis

**FIGURE 6:**
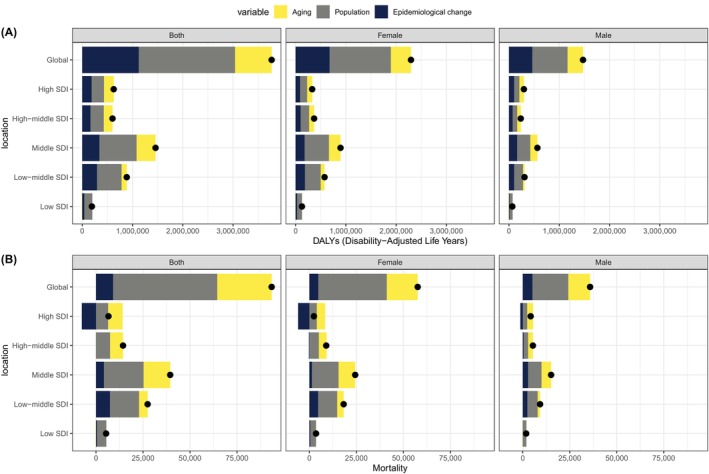
Decomposition analysis of DALYs and mortality due to T2DM attributed to LPA worldwide, 1990–2021. (A) Changes in global and sociodemographic index (SDI) quintile‐specific DALYs for T2D attributed to low physical activity. (B) Changes in global and SDI quintile‐specific mortality rates for T2D attributed to low physical activity. DALYs, disability‐adjusted life years; LPA, low physical activity; SDI, sociodemographic index; T2DM, type 2 diabetes mellitus.

Our study used decomposition analysis (Figure 6) delineated the complex influence of aging, population growth, and epidemiological changes on both DALYs (Table S2) and mortality (Table S3) across SDI and genders. From 1990 to 2021, the DALYs of T2DM attributed to LPA was primarily driven by population growth (50.84%), epidemiological changes (29.84%), and aging (19.33%) (Table S2). The mortality rate for T2DM attributable to LPA was increased by population growth (59.3%), followed by aging (30.99%) and epidemiological changes (9.71%) (Table S3). Population growth stood out as the most substantial contributor to the burden of T2DM, with its impact ranging from 39.41% in high SDI quintiles to a striking 82.7% in low SDI quintiles, emphasizing the pervasive effect of demographic expansion on T2DM.

### 3.5 Association Between SDI Values and ASMR, ASDR, and PAFs

**FIGURE 7:**
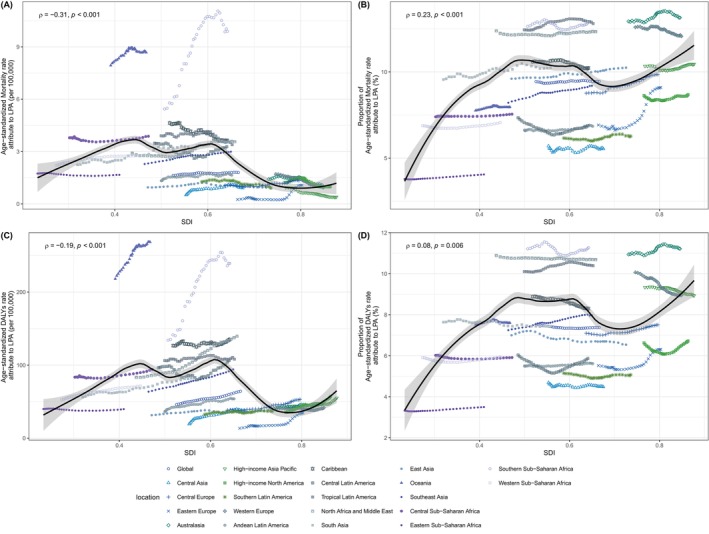
The relationship between disease burden of T2DM attribute to LPA and SDI values. (A) ASMR and SDI in 2021 by GBD regions, (B) PAFs in ASMR and SDI in 2021 by GBD regions. (C) ASDR and SDI in 2021 by GBD regions, (D) PAFs in ASDR and SDI in 2021 by GBD regions. ASDR, age‐standardized disability‐adjusted life years rate; ASMR, age‐standardized mortality rate; PAFs, population attributable fractions; LPA, low physical activity; SDI, sociodemographic index; T2DM, type 2 diabetes mellitus.

The ASMR for T2DM attributable to LPA exhibited an M‐shaped relationship with SDI values globally (Figure 7). The ASMR increased initially, peaking at an SDI value of approximately from 0.43 to 0.60, and then decreased as SDI values continue to rise. A significant negative correlation existed between SDI and both ASMR and ASDR, with correlation coefficients (*ρ*) of −0.31 (*p* < 0.001) and −0.19 (*p* < 0.001), respectively. This indicates that higher SDI values are associated with a lower burden of T2DM.

However, the PAFs for both ASMR and ASDR display a different trend, correlating positively with SDI and leveling off within the range of 0.5–0.7, with *ρ* values of 0.23 (*p* < 0.001) and 0.08 (*p* = 0.006). These findings imply that while the disease burden is better managed in regions with higher SDI, the prevalence of LPA, a key risk factor for T2DM, may paradoxically be more pronounced in these areas and contributed more to the ASMR.

## 4 Discussion

Our study provided a comprehensive update on the global burden of T2DM attribute to LPA over the period from 1990 to 2021 encompassing data from 204 countries. Our findings indicated a substantial increase in the global DALYs and deaths for T2DM attributable to LPA, with significant variations observed by gender, SDI, and region. From 1990 to 2021, the global burden of disease for T2DM due to LPA increased significantly, about an approximately 3.13‐fold increase. ASDR and ASMR was on the rise, was growing faster in men than women, which might be caused by factors health services, combined with cultural and social barriers faced by women. Population growth was the largest contributor to the burden of T2DM, while epidemiological changes also played an important role in DALYs and mortality. In addition, our study suggested that LPA might be more prevalent and had a greater health impact on T2DM in areas with higher SDI. Our findings had important implications for public health, emphasizing the need for interventions aimed at increasing PA levels to reduce the burden of T2DM. Our results were consistent with existing literature highlighting the role of LPA in the development of T2DM and provided a more nuanced understanding of the epidemiological trends and demographic patterns [6].

The mortality risk in diabetes mellitus (DM) adults are lower than that in the non‐DM patients. PA has been reported as an indispensable factor in lowering or even canceling out this excess mortality risk [22]. Decreased cardiorespiratory endurance due to insufficient PA is one of the major influencing factors for the occurrence, development, and prognosis of T2DM, and is highly correlated with increased mortality of CVD, all‐cause mortality, and multiple cancers [23, 24, 25]. LPA may lead to increased insulin resistance because insufficient activity reduces the uptake and utilization of glucose by muscles [26]. In addition, LPA may also affect the distribution and function of adipose tissue and increase abdominal fat, which is related to insulin resistance [27]. Any type of PA can improve metabolic abnormalities, enhances islet beta cell function, regulates blood vessel function, and improves the gut microbiome, all of which contribute to better health management and reduced disease risk [28, 29, 30]. Exercise can increase the time of glucose reaching the target, reduce the daytime glucose fluctuation, significantly increase the glucose in range (TIR) of diabetic patients and does not significantly increase hypoglycemia [31]. One study suggested that increasing moderate to vigorous PA in place of sedentary behavior or light PA might help improve sleep quality, which also improved type 2 diabetes [32]. In summary, PA is essential for maintaining normal glucose metabolism.

The gender differences in ASDR and ASMR suggested that although both men and women were affected by LPA, the rate was bigger in women and the rate of increase was faster in men. This might be related to gender‐specific behavioral patterns, social roles, and differences in response to health promotion messages. In addition, women had higher PAF in areas with high SDI, which reflected that women were more sensitive to increased PA in these areas, or that female population was more effectively targeted in health promotion strategies. There was a cross‐sectional study design to explore the pooled time spent on PA among adolescents across multiple regions between 2003 and 2019 [33]. Prior to adolescence, young men and women had similar aerobic capacity, strength, body composition, and overall athletic performance [34]. So, we could see there was no difference between men and women in children in APC model in our study. The extent of sex differences increases as individuals mature [35]. There are average differences in aerobic capacity, body composition, and strength between adolescent and adult males and females [36]. After puberty, males have stronger aerobic capacity than females due to changes such as increased hemoglobin and thinner body composition [36]. In our study, we could see the same inclusion that there were no obvious abnormalities at the lower age, but the changes appeared more obvious as the age increased.

The PA level of T2DM patients is generally low, which is related to modern lifestyle, urbanization, sedentary, and other factors. Compared with rural residents, urban residents have lower PA level, and the rapid process of aging society makes a large number of elderly patients further decline in PA level due to diseases, decline in physical function and social environmental restrictions [37]. The relationship between SES and disease burden reveals deep‐seated problems with health inequalities [38]. Countries with low SDI increased rapidly in our study, face greater challenges, which may be related to limited resources, difficulty in implementing health promotion strategies, and low awareness of the importance of PA. SES may affect an individual's PA level and risk of T2DM. The low SES population may face more obstacles, such as a lack of safe sports venues, time, and resource limitations, which may lead to an increased risk of LPA and T2DM [39]. Conversely, countries with high SDI, while performing better in disease management, decreased the burden of T2DM, suggesting the need for more targeted interventions in these areas. As the global population ages, significant regional differences in the aging process profoundly impact disease incidence and burden [40]. Notably, the rate of aging varies globally, potentially leading to an imbalance in disease burden across regions. High SDI regions typically have longer life expectancy and a faster aging process, resulting in a more significant burden of T2DM among the elderly, like Eastern Europe, which displayed most significant increase. Conversely, although low SDI and low‐middle SDI regions experience a relatively slower rate of aging, insufficient health resources, and limited health services result in poor disease management and treatment outcomes among the elderly, maintaining a high disease burden. The application of the APC model provides us with an in‐depth understanding of age, period, and cohort effects. Population growth is the main driver of the increased burden of T2DM, which underscores the importance of controlling population growth and improving population health. At the same time, the influence of aging on DALYs and mortality is complex and changeable, and we need to adopt multifaceted strategies to deal with it. The decomposition analysis further highlights the combined impact of population growth, aging, and epidemiological changes on the burden of T2DM. The interaction of these factors suggests that a single intervention may not be sufficient to effectively control the growth of T2DM, and a combination of factors needs to be considered [41]. Patients have different responses to PA, which may be related to biological differences, sociocultural factors, and health behavior patterns.

During the study period, the worldwide outbreak of COVID‐19 between 2020 and 2021 may have significantly influenced the levels of PA and mortality rates of T2DM [42, 43]. The pandemic led to widespread lockdowns, social distancing measures, and disruptions in healthcare services, which could have contributed to reduced PA levels and increased mortality rates. It is important to note that these factors may have exacerbated the burden of T2DM attributed to LPA, potentially leading to an underestimation of the true impact of LPA in the absence of the pandemic. Furthermore, the pandemic's impact on healthcare systems worldwide may have disproportionately affected T2DM populations. The disruption in routine diabetes care and management could have contributed to worsening health outcomes and increased mortality. It is crucial for future studies to consider the distinct effects of the pandemic to accurately attribute the burden of T2DM to LPA and other risk factors.

## 5 Conclusion

This study delineated the burden and trends of T2DM attributable to LPA. LPA has inflicted a substantial disease burden on the global population, predominantly in middle‐aged and elderly groups. These results underscore the importance of identifying LPA as a modifiable risk factor for T2DM. This study has demonstrated the significant burden of T2DM attributable to LPA, with careful consideration given to the exclusion of CVD in mortality figures. They provide crucial guidance for crafting impactful public health strategies and policies to counteract the escalating mortality toll of this condition. Global health policies must also take full account of gender differences to ensure women's equitable access to health services.

## 6 Limitations

Nonetheless, this study faces several constraints. Firstly, some data in this study rely on estimates, which have uncertainties. Secondly, this study overlooks comorbidities and disease complexities, suggesting a need for further refinement and investigation. Thirdly, the COVID‐19 pandemic between 2020 and 2021 may have significantly influenced the mortality of T2DM, and our analysis may not fully explore the true impact of LPA during this period. Future research should aim to disentangle the effects of the pandemic from the long‐term trends in T2DM burden.

## Author Contributions

Lihang Yang, Diya Xie, and Fengmin Liu contributed equally to this manuscript. Lihang Yang, Diya Xie, and Fengmin Liu contributed in data analysis and manuscript writing. Kun Zhang conceptualized and designed these studies, performed them, and supervise the program. Jiaying Lin, Xin Lin, and Yuquan Chen contributed in data acquisition. All authors contributed to the manuscript revision, and read and approved the submitted version.

## Ethics Statement

For the use of deidentified data in GBD study, a waiver of informed consent has been approved by the University of Washington Institutional Review Board.

## Consent

Given that the research solely entailed data analysis from the GBD 2021 study and did not involve human subjects, the need for informed consent and institutional review board approval was waived.

## Conflicts of Interest

The authors declare no conflicts of interest.

## Supporting information


Data S1.

## Data Availability

The datasets analyzed during the current study are available in the GBD 2021 online repository (http://ghdx‐health‐data‐org/gbd‐results‐tool).
